# Systematic druggable genome-wide Mendelian randomization identifies therapeutic targets for lung cancer

**DOI:** 10.1186/s12885-024-12449-6

**Published:** 2024-06-04

**Authors:** Wenfu Song, Yingying Li, Yaxuan Yao, Shiling Sun, Xutao Guan, Bing Wang

**Affiliations:** 1 Department of Hematology and Oncology, the First Affiliated Hospital of Henan University of Chinese Medicine, Zhengzhou, Henan Province China; 2 The First Clinical Medical College of Henan University of Chinese Medicine, Zhengzhou, Henan Province China

**Keywords:** Lung cancer, Druggable target, Mendelian randomization, Colocalization, Gene expression

## Abstract

**Background:**

Drug repurposing provides a cost-effective approach to address the need for lung cancer prevention and therapeutics. We aimed to identify actionable druggable targets using Mendelian randomization (MR).

**Methods:**

Summary-level data of gene expression quantitative trait loci (eQTLs) were sourced from the eQTLGen resource. We procured genetic associations with lung cancer and its subtypes from the TRICL, ILCCO studies (discovery) and the FinnGen study (replication). We implemented Summary-data-based Mendelian Randomization analysis to identify potential therapeutic targets for lung cancer. Colocalization analysis was further conducted to assess whether the identified signal pairs shared a causal genetic variant.

**Findings:**

In the main analysis dataset, we identified 55 genes that demonstrate a causal relationship with lung cancer and its subtypes. However, in the replication cohort, only three genes were found to have such a causal association with lung cancer and its subtypes, and of these, HYKK (also known as AGPHD1) was consistently present in both the primary analysis dataset and the replication cohort. Following HEIDI tests and colocalization analyses, it was revealed that HYKK (AGPHD1) is associated with an increased risk of squamous cell carcinoma of the lung, with an odds ratio and confidence interval of OR = 1.28,95%CI = 1.24 to 1.33.

**Interpretation:**

We have found that the HYKK (AGPHD1) gene is associated with an increased risk of squamous cell carcinoma of the lung, suggesting that this gene may represent a potential therapeutic target for both the prevention and treatment of lung squamous cell carcinoma.

**Supplementary Information:**

The online version contains supplementary material available at 10.1186/s12885-024-12449-6.

## Introduction

Lung cancer is the leading cause of cancer death worldwide [1], with more than one million deaths each year [2]. According to tissue type, lung cancer can be divided into small cell lung cancer (SCLC) and non-small cell lung cancer (NSCLC),among which NSCLC mainly includes lung adenocarcinoma and lung squamous cell carcinoma [3]. The pathogenesis of lung cancer is a complex process involving various risk factors [4]. At present, the main treatment methods of lung cancer include surgery, chemotherapy, radiation therapy and biological therapy.

However, the treatment of lung cancer still faces huge challenges. Recurrence and metastasis after surgery will still lead to disease progression, and chemotherapy drugs in the treatment of tumors are accompanied by a large number of toxic side effects, such as bone marrow suppression, hair loss, bleeding, liver and kidney function injury [5, 6]. At present, the treatment mode of lung cancer has gradually shifted to targeted therapy, and there are different therapeutic drugs for different molecular targets [7]. However, targeted therapy still has bottlenecks: On the one hand, targeted drugs are ineffective without driver gene mutation,On the other hand, after a median treatment time of 12 to 14 months, most drugs develop acquired resistance, leading to disease progression [8]. Therefore, finding new therapeutic targets for lung cancer is still the focus and difficulty of research.

Large-scale human genetics studies provide an opportunities for the development of new drugs for many sophisticated diseases, as drug targets supported by genetic evidence have a greater likelihood of success during drug development [9, 10]. In short, “medicable” genes that code for protein or gene expression can afford thread to find drug targets [11]. Currently, many large-scale genome-wide association studies (GWASs) have identified many single nucleotide polymorphisms (SNPs) associated with lung cancer risk, however, because many of the identified SNPS are located in non-coding regions or gene intervals, GWAS data do not provide clear and direct clues about disease-causing genes and drug targets.

Mendelian randomization (MR) analyses use inherited variation as an instrumental variable to enhance inferences of causality between exposure and outcomes. This approach is less susceptible to confusion and reverse causality bias than observational studies because genetic variation is randomly distributed at conception and is not modified by acquired factors and disease onset [12]. Today, MR analysis has been diffusely used to re-use confirmed drugs and to identify new remedial targets by coordinating aggregated data from disease GWAS and quantitative trait loci (eQTL) studies [13–15]. The level of expression of a gene can be well thought out a lifetime exposure, and eQTL located in genomic regions of the medicable gene is often considered a proxy [16, 17]. In this study, we implemented a systematic pharmacizable genome-wide SMR to identify therapeutic targets for lung cancer and its subtypes.

## Methods

**Fig. 1:**
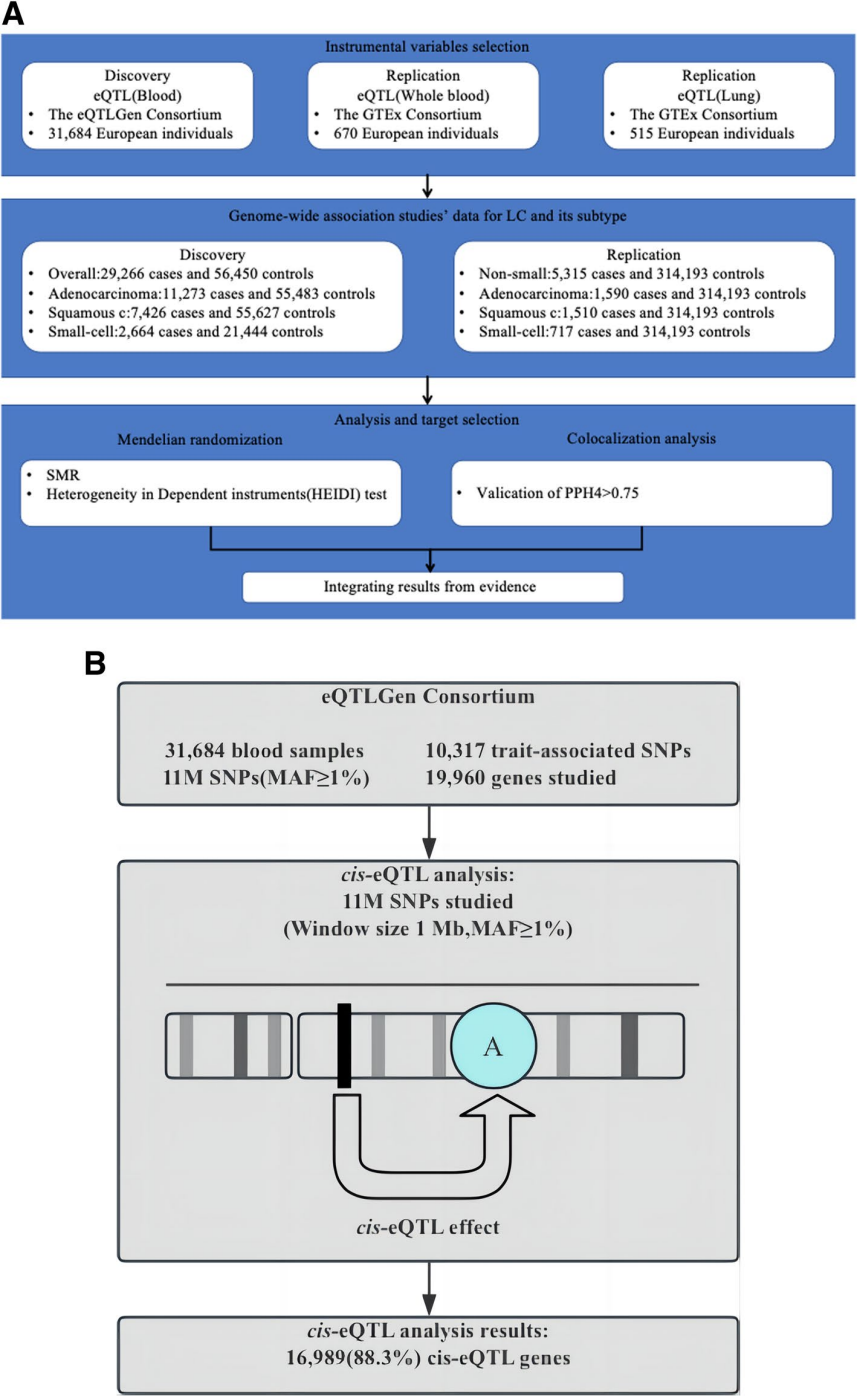
**A** Study design. SMR, summary-based Mendelian randomization; QTL, quantitative trait loci; LC, Lung Cancer; PPH4, posterior probability of H4. **B** cis-eQTL analysis

Figure 1A shows the overall design of the study. In this study, we used data sets from the Transdisciplinary Research for Cancer of Lung (TRICL) and The International Lung Cancer Consortium (ILCCO) as the primary analysis, involving 29,266 patients of European descent and 56,450 controls [18]. To verify our findings, we used the FinnGen study dataset for replication. The discovery eQTL dataset was procured from eQTLGen (https://eqtlgen.org/), in which eQTLs for 16,987 genes were procured from 31,684 blood samples from individuals of healthy European ancestry [19]. Meanwhile, as surrogate data for eQTL analyses, we utilized human lung tissue and whole blood samples from the GTEx Consortium, comprising 515 and 670 samples, respectively [20].

Figure 1B show the cis-eQTL analysis. The original eQTL data consisted of 31,684 blood samples, 10,317 trait associated SNPs, 11 M SNPs (MAF ≥ 1%) and 19,960 genes studied. We selected data with a window size of 1 Mb and a MAF ≥ 1%, resulting in 16,989 (88.3%) cis-eQTL genes. The data processing methods for GTEx eQTLs are consistent with those of eQTLGen consortium.

### Lung cancer outcome datasets summary

Data for lung cancer and its subtypes were obtained from the TRICL, ILCCO studies, and the FinnGen study. In the TRICL and ILCCO studies, all participants were of European ancestry, comprising 29,266 European-ancestry patients and 56,450 controls, with 11,273 cases and 55,483 controls for lung adenocarcinoma,7,426 cases and 55,627 controls for lung squamous cell carcinoma, and 2,664 cases and 21,444 controls for small cell lung cancer [18]. Summary data on the genetic associations of lung cancer and its subtypes were derived from the publicly available R10 data of the FinnGen study, which included 5,315 cases and 314,193 controls for non-small cell lung cancer, 1,590 cases and 314,193 controls for lung adenocarcinoma, 1,510 cases and 314,193 controls for lung squamous cell carcinoma, and 717 cases and 314,193 controls for small cell lung cancer [21].

### Summary-data-based MR analysis

A summary-data-based Mendelian Randomization (SMR) approach was employed to identify associations between systemic “druggable” gene eQTL data and the risk of lung cancer and its subtypes [17]. SMR can achieve higher statistical power than classical MR analyses when exposure and outcome are obtainable from two independent samples with large sample sizes based on top associated cis-QTLs [17]. Cis-eQTLs were selected within a ± 1000 kb window around the corresponding genes using a *P*-value threshold of 1.0 × 10^−5^. SNPs with allele frequency differences greater than a specified threshold (set at 0.01 in this study) among any pair of datasets (including LD reference samples, QTL summary data, and outcome summary data) were excluded. The heterogeneity in dependent instruments (HEIDI) test was applied to distinguish pleiotropy from linkage, where P-HEIDI < 0.05 was considered potentially due to pleiotropy and thus discarded from the analysis. SMR and HEIDI tests were performed using the SMR software tool (SMR v1.3.1,the code is available at https://yanglab.westlake.edu.cn/software/smr/#Overview). The *P*-values was adjusted by Benjamini-Hochberg method, and to control the false discovery rate (FDR) at 0.05. Co-localization analysis was conducted on results with FDR-corrected *P*-values < 0.05 and P-HEIDI > 0.05.

### Colocalization analysis

Colocalization analysis was carried out using the coloc R package to investigate the potential causal relationship between systemic “druggable” genes and lung cancer and its subtypes [22]. There are four primary hypotheses in colocalization analysis: H0: SNPs at the selected locus are unrelated to both exposure (eQTL) and disease (lung cancer). H1: SNPs at the selected locus are related to exposure (eQTL) but not to disease (lung cancer). H2: SNPs at the selected locus are related to disease (lung cancer) but not to exposure (eQTL). H3: SNPs at the selected locus are related to either exposure (eQTL) or disease (lung cancer), but these are distinct SNPs (LD). H4: SNPs at the selected locus are related to both exposure (eQTL) and disease (lung cancer), sharing the same SNP (LD). A PPH4 value > 0.75 for H4 is considered evidence supporting colocalization [23].

## Results

### SMR analysis of cis-eQTLs and lung cancer and its subtype

**Fig. 2:**
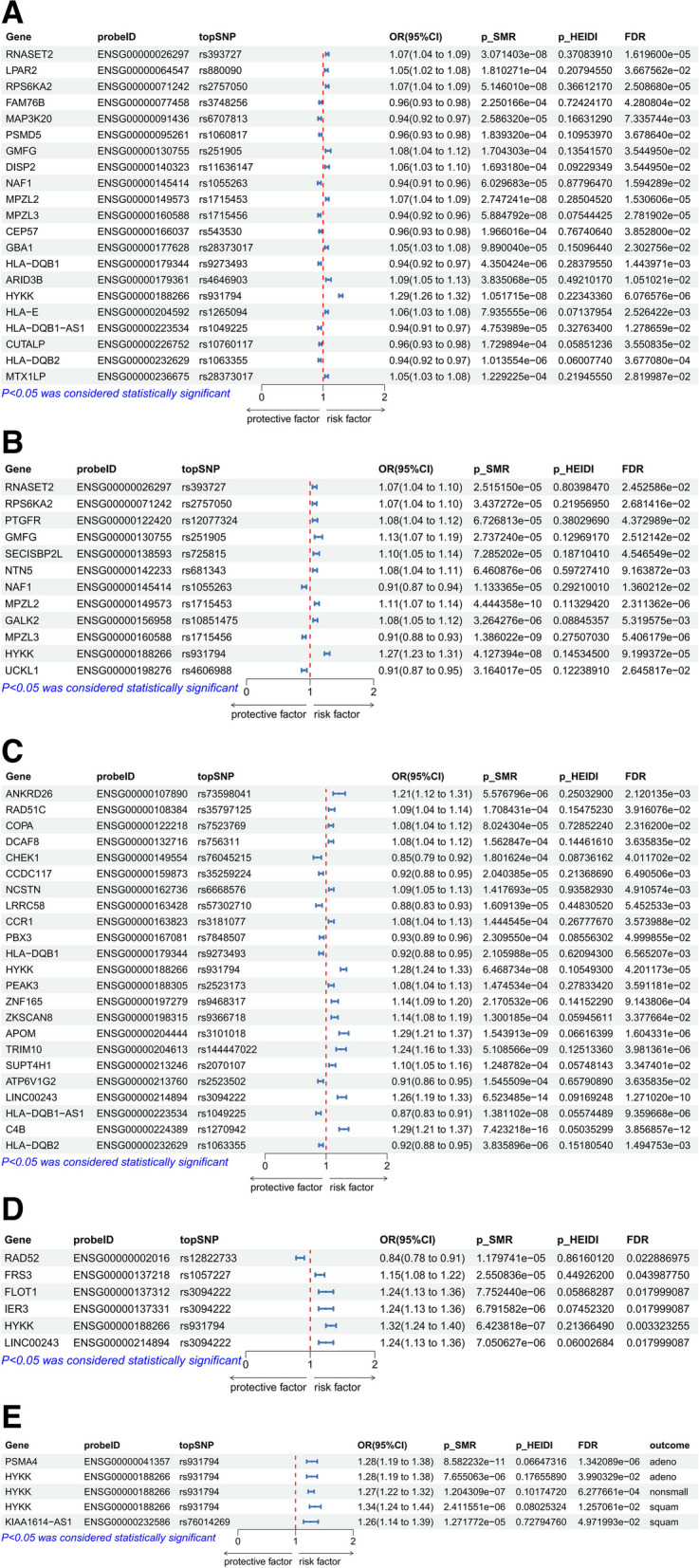
**A** Associations of predicted gene expression with lung cancer in Mendelian randomization analysis. **B** Associations of predicted gene expression with lung adenocarcinoma cancer in Mendelian randomization analysis. **C** Associations of predicted gene expression with lung squamous cell carcinoma cancer in Mendelian randomization analysis. **D** Associations of predicted gene expression with small cell lung cancer in Mendelian randomization analysis. **E** Associations of predicted gene expression with lung cancer (replication) in Mendelian randomization analysis. OR, odds ratio; CI, confidence interval

In the main analysis dataset results, the causal relationships between blood-derived eQTLs and lung cancer and its subtypes are depicted in forest plots (Fig. 2A through E). After excluding SNPs with P-HEIDI < 0.05 and FDR-adjusted *P*-values > 0.05 via SMR analysis, we identified a total of 21 genes that have a causal relationship with lung cancer, including RNASET2, LPAR2, RPS6KA2, FAM76B, MAP3K20, PSMD5, GMFG, DISP2, NAF1, MPZL2, MPZL-3, CEP57, GBA1, HLA-DQB1, ARID3B, HYKK (AGPHD1), HLA-E, HLA-DQB1-AS1, CUTALP, HLA-DQB2, and MTX1LP; these findings can be found in detail in Fig. 2A. There were 12 genes with a causal relationship to lung adenocarcinoma, including RNASET2, SECISBP2L, RPS6KA2, PTGFR, MPZL3, GMFG, NTN5, NAF1, MPZL2, GALK2, HYKK (AGPHD1), and UCKL1, as shown in Fig. 2B. For lung squamous cell carcinoma, 23 genes were causally related, comprising ANKRD26, RAD51C, PEAK3, PBX3, COPA, DCAF8, CHEK1, CCDC117, NCSTN, LRRC58, CCR1, HYKK (AGPHD1), HLA-DQB1, ZNF165, HLA-DQB1-AS1, TRIM10, SUPT4H1, C4B, HLA-DQB2, ATP6V1G2, APOM, LINC00243, and ZKSCAN8, as detailed in Fig. 2C. Six genes were found to have a causal relationship with small cell lung cancer, namely RAD52, FRS3, FLOT1, IER3, HYKK (AGPHD1), and LINC00243, which are illustrated in Fig. 2D.

In the replication cohort results, we observed that PSMA4 has a causal relationship with lung adenocarcinoma, KIAA1614-AS1 is causally linked to lung squamous cell carcinoma, and HYKK (AGPHD1) has a causal relationship with non-small cell lung cancer, lung adenocarcinoma, and lung squamous cell carcinoma; these findings are presented in Fig. 2E. Notably, only HYKK (AGPHD1) was replicated in the FinnGen study, and it was consistently replicated across non-small cell, adenocarcinoma, and squamous subtypes.

### SMR analysis of replication cis-eQTLs and lung cancer and its subtype

Results from the primary analysis dataset using GTEx whole blood and lung cancer and its subtypes. After excluding SNPs with P-HEIDI < 0.05 and FDR-adjusted *P*-values > 0.05 via SMR analysis, we identified a total of 18 genes that have a causal relationship with lung cancer, including U919328.19, GABBR1, IER3, LINC00243, SKIV2l, HLA-DQA1, HLA-DQB1, HLA-DQB1-AS1, HLA-DQB2, RNASET2, RP1-167A14.2, PSMD5-AS1, MPZL2, MPZL3, PSMA4, UCKL1 and PRPF6. There were 5 genes with a causal relationship to lung adenocarcinoma, including RNASET2, MPZL2, MPZL3, CHRNA5 and UCKL1. For lung squamous cell carcinoma, 11 genes were causally related, comprising COPA, NCSTN, U91328.19, GABBR1, IER3, HLA-DQB1-AS1, HLA-DQB2, ANKRD26, PKD2L1, PSMA4 and RAD51C. Only one gene was found to have a causal relationship with small cell lung cancer, namely PSMA4. However, in the replication cohort results, we only observed that PSMA4 has a causal relationship with lung adenocarcinoma. Thus, the causal relationship between PSMA4 and lung cancer, as well as its subtypes, requires further investigation.

Results from the primary analysis dataset using GTEx human lung tissue and lung cancer and its subtypes. After excluding SNPs with P-HEIDI < 0.05 and FDR-adjusted *P*-values > 0.05 via SMR analysis, we identified a total of 14 genes that have a causal relationship with lung cancer, including FUBP1, RP11-218F10.3, CTD-2012J19.1, BTN2A2, TRIM31, FLOT1, LINC00243, HLA-DQA1, HLA-DQB1, HLA-DQB1-AS1, HLA-DQB2, RNASET2, AC011330.5 and SECISBP2L. There were 6 genes with a causal relationship to lung adenocarcinoma, including FUBP1, CTD-2012J19.3, CTD-2012J19.1, DCBLD1, NRG1 and SECISBP2L. For lung squamous cell carcinoma, 9 genes were causally related, comprising U91328.19, FLOT1, LINC00243, HLA-DQA1, HLA-DQB1, HLA-DQB1-AS1, HLA-DQB2, ZNF483 and BLOC1S2. They are not found to have a causal relationship with small cell lung cancer. In the replication cohort results, We have not identified any genes that show a causal association with lung cancer or its subtypes. Therefore, further research is needed to elucidate the causal relationships between eQTLs in human lung tissue and lung cancer, including its various subtypes.

### Colocalization analysis

In the results of the SMR analysis, we discovered that HYKK (AGPHD1) was consistently identified in lung cancer data from both TRICL and ILCCO studies, and it was replicated specifically in non-small cell lung cancer, lung adenocarcinoma, and lung squamous cell carcinoma within the FinnGen dataset. To substantiate these findings, colocalization analysis was performed to estimate the posterior probability of common variants being causally associated with the disease outcomes. For the significant MR results, we retrieved all SNPs located within a ± 100 kb window upstream and downstream of each SNP for colocalization analysis between eQTLs and GWAS hits. In the main analysis dataset results, HYKK (AGPHD1) showed 100% posterior probability of shared causal variant (PP.H4 = 100%) across all four subtypes considered. In the replication cohort results, HYKK (AGPHD1) displayed varying degrees of colocalization evidence: for the non-small subtype, PP.H4 was 28.5%; for the lung adenocarcinoma subtype, PP.H4 was 35.2%; and for the lung squamous cell carcinoma subtype, PP.H4 was 98.9% (indicating strong evidence for colocalization since PP.H4 > 0.75). These detailed colocalization probabilities are presented in Table 1. This suggests that HYKK (AGPHD1) is a promising candidate gene for further investigation into its role as a potential causal factor in multiple subtypes of lung cancer, particularly in lung squamous cell carcinoma, based on the high degree of colocalization likelihood observed. The colocalization results for the remaining genes are presented in Table S2.

**Table 1:** Results of colocalization of HYKK (AGPHD1) with lung cancer and its subtypes

Outcome	PP.H0	PP.H1	PP.H2	PP.H3	PP.H4	PP for shared variant
Discovery						
Over all	1.83e-96	3.64e-06	5.04e-94	4.15e-06	1.00	100%
Adenocarcinoma	1.44e-44	3.64e-06	3.98e-42	5.01e-06	1.00	100%
Squamous	2.61e-37	3.64e-06	7.19e-35	4.96e-06	1.00	100%
Small cell	5.79e-19	3.65e-06	1.60e-16	7.35e-06	1.00	100%
Replication						
Non small	7.31e-27	2.58e-03	2.02e-24	7.12e-01	2.85e-01	28.5%
Adenocarcinoma	4.14e-06	2.34e-03	1.14e-03	6.45e-01	3.52e-01	35.2%
Squamous	1.28e-13	4.22e-05	3.54e-11	1.06e-02	9.89e-01	98.9%

### Integrating evidence

**Fig. 3:**
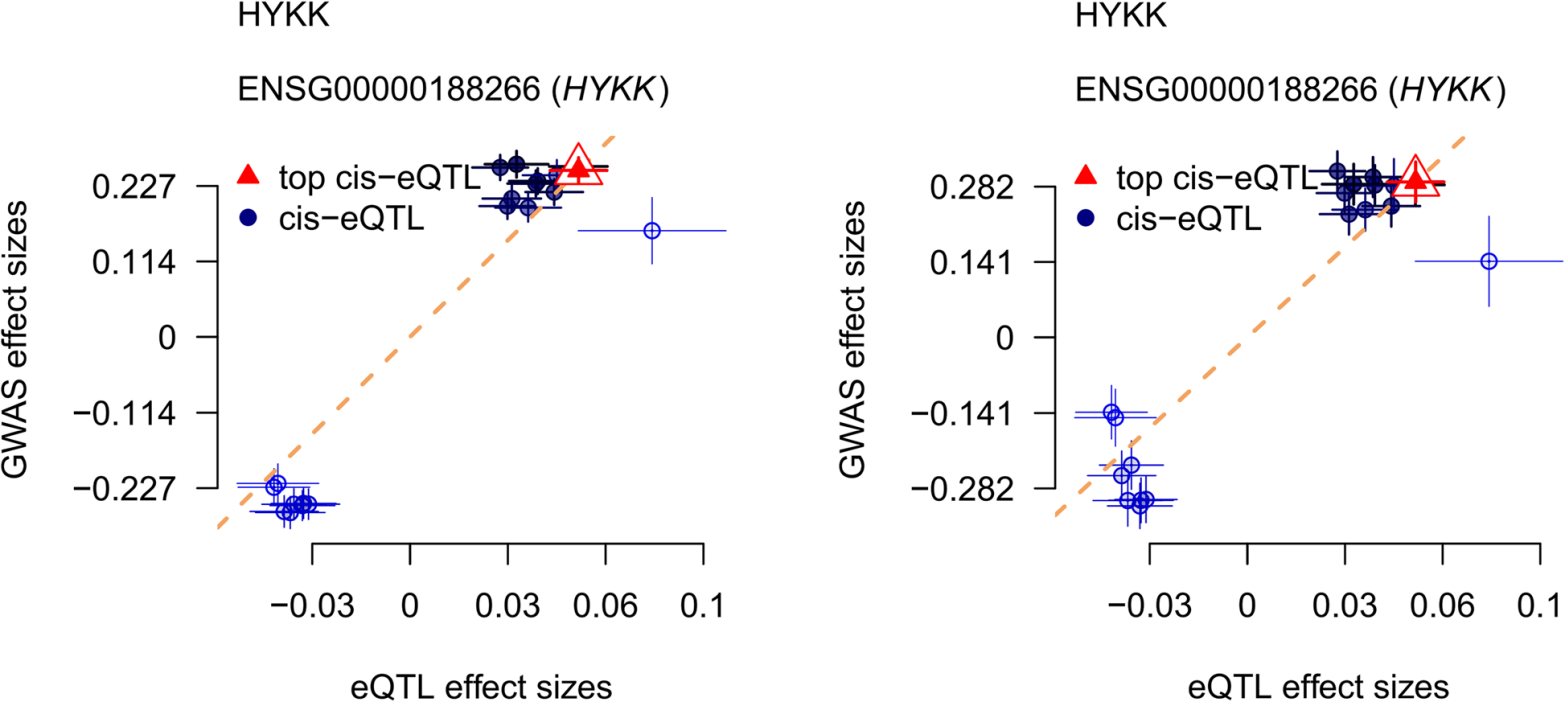
Scatter plot for associations between HYKK (AGPHD1) gene and lung squamous cell carcinoma

After combining the results of different methods, we finally found a potential causal association between HYKK (AGPHD1) and lung squamous cell carcinoma. In order to clarify the causal relationship between HYKK (AGPHD1) and lung squamous cell carcinoma, a conditional Logistic regression model was used to calculate the OR value and 95%CI of lung squamous cell carcinoma associated with the use of candidate drugs, and the results were visualized by YangLab SMR visualization method (Fig. 3). The results showed that HYKK (AGPHD1) was associated with an increased risk of lung squamous cell carcinoma in the primary analysis set (OR: 1.28; 95% CI: 1.24–1.33), which is consistent with the results in the replication queue (OR: 1.34; 95% CI: 1.24–1.44; Fig. 2C and E).

**Fig. 4:**
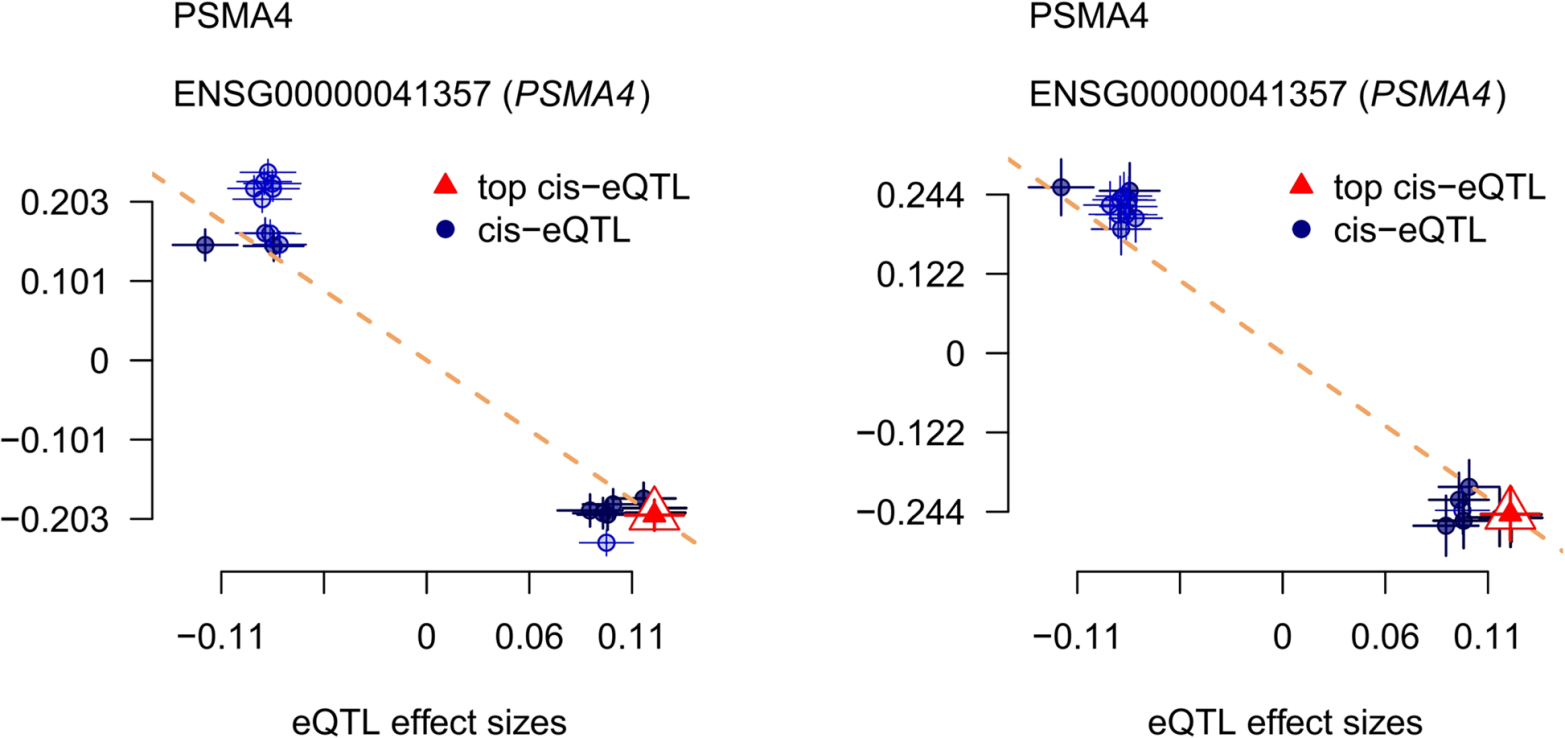
Scatter plot for associations between PSMA4 gene and lung adenocarcinoma

Indeed, it’s intriguing that while a causal link between PSMA4 and lung adenocarcinoma was not evident in the primary datasets, replication cohorts from eQTLGen and GTEx analyses involving whole blood tissue have suggested a potential causal relationship. Utilizing the YangLab SMR visualization approach has facilitated the illustration of these findings, pointing towards PSMA4 potentially acting as a protective factor against lung adenocarcinoma (Fig. 4). Nonetheless, additional studies are imperative to confirm this causal relationship and to ascertain the exact nature and magnitude of its protective effect. Further research should involve more granular analyses, larger sample sizes, and possibly experimental models to validate the observed association and explore the underlying biological mechanisms.

## Discussion

In an effort to identify opportunities for drug repurposing and development targeting lung cancer and its subtypes, we conducted a large-scale MR analysis that leverages the underlying genetic mechanisms of gene expression as robust evidence. Our study identified hydroxylysine kinase (HYKK), also known as amino-glycoside phosphotransferase 1 (AGPHD1), as a potentially causally related druggable target gene for squamous cell carcinoma of the lung.

In our research, we found that rs931794 in the HYKK (AGPHD1) gene is a risk factor for lung squamous cell carcinoma. However, Y. Bossé et al. utilized human lung tissue eQTL and lung cancer GWAS data, employing TWAS analysis through S-PrediXcan and FUSION methods, which identified HYKK (AGPHD1) as a protective factor for lung cancer, a finding that differs from the results of our study [24]. It should be noted that genes uncovered by TWAS analyses do not necessarily imply causality,rather, TWAS analysis are more aptly interpreted as prioritized or ordered candidate causal genes at specific loci. Furthermore, TWAS analyses are unable to distinguish causality from pleiotropy. Consequently, in the study by Y. Bossé et al., no definitive conclusions were drawn regarding the relationship between HYKK (AGPHD1) and either lung cancer in general or specifically lung squamous cell carcinoma, and the replication data cohort within their investigation failed to replicate the reported association between HYKK and lung cancer. Thus, the study by Y. Bossé et al. is not contradictory to our research, but rather, our investigation employs the use of the SMR analysis, which offers a stronger assessment of causal relationships compared to TWAS methodologies.

Moreover, numerous studies align with the findings of our investigation. Wang Hong et al. conducted a meta-analysis using STATA software incorporating nine studies and suggested that the rs8034191 variant in the HYKK (AGPHD1) gene might not alter the genetic risk for lung cancer in Asian populations [25]. However, multiple meta-analyses have shown that the rs8034191 variant in the HYKK (AGPHD1) gene is a risk factor for lung cancer, with individuals carrying the HYKK (AGPHD1) rs8034191 (A > G) variant having a higher likelihood of developing lung cancer [26–29]. Qi Wang through case-control studies involving 611 cases and a meta-analysis, established a relationship between the rs931794 variant and increased lung cancer risk in the Chinese population, with stratified analysis revealing a significant association between the G allele and lung cancer risk among smokers [30].

We propose that HYKK (AGPHD1) may function as a disease-causing gene specifically for lung squamous cell carcinoma, although the exact physiological role of HYKK (AGPHD1) remains unclear. Qi Wang discovered that the HYKK (AGPHD1) gene interacts with the CHRNA5-CHRNA3-CHRNB4 cluster and is implicated in the risk of lung cancer development [30]. The CHRNA5-CHRNA3-CHRNB4 cluster encodes nicotinic acetylcholine receptor subunits primarily expressed in aspiratory epithelium, pneumonic neuroendocrine cells, and lung cancer cells [31]. Studies have shown that these receptors can bind to nicotine, N’-nitrosonornicotine, and other potential carcinogens, leading to gene mutations and tumor formation [32]. Furthermore, Tournier found that the injury-induced acetylcholine receptors regulate intracellular calcium ions in the process of respiratory epithelial wound repair [33], while Krais demonstrated that nicotinic acetylcholine receptors play a crucial role in tumorigenesis by regulating adhesion and motility of respiratory epithelial cells [34]. Therefore, we hypothesize that SNPs within the CHRNA5-CHRNA3-CHRNB4 cluster could affect subjective cancer risk by altering receptor nicotine binding, normal cell proliferation, cell migration, and wound healing processes. Similarly, HYKK (AGPHD1) may also potentially influence the risk of developing lung squamous cell carcinoma through this same pathway.

The primary strengths of this study lie in the use of Summary-data-based MR analysis and Colocalization analysis, which together utilize genetic variations to estimate causal effects between gene expression and lung cancer risk. The SMR and MR analyses both utilize SNPs as instrumental variables, thereby minimizing confounding factors and biases from reverse causality, thus providing a more accurate assessment of the causal relationship between exposures and outcomes. The difference lies in the fact that SMR utilizes QTLs (quantitative trait loci, such as gene expression levels, protein expression levels, etc.) as exposures. Since genes and proteins are upstream elements in the central dogma and constitute an inherent process in the development of all diseases, SMR inherently possesses greater computational power when identifying drug targets compared to MR. Moreover, SMR effectively performs colocalization analysis as well. Secondly, we employed HEIDI tests and colocalization methods to mitigate biases due to linkage disequilibrium and horizontal pleiotropy. SMR is also equivalent to performing colocalization analysis, but unlike other colocalization methods, it can simultaneously assess the causal relationship between exposure and outcome. Therefore, by utilizing both SMR and colocalization methods, our conclusions become even more robust. Thirdly, the GWAS datasets used in our main analysis had large sample sizes, enhancing the statistical power of our study. Fourthly, replication across different datasets provided additional support for our findings. Moreover, to minimize bias due to various genetic backgrounds, we restricted our sample selection to individuals of European ancestry. Lastly, by leveraging comprehensive gene expression datasets, we adopted a stringent selection process to evaluate actionable drug targets and uncovered HYKK (AGPHD1) as a treatment target associated with the risk of lung squamous cell carcinoma.

However, our study has certain limitations. Firstly, the eQTL dataset we used was insufficient to cover the entire druggable genome, lacking data on gene expression genetic variations related to the X and Y chromosomes. Secondly, we only utilized cis-eQTL instrumental variables, which minimizes the potential bias from horizontal pleiotropy but may limit our ability to detect additional potential causal associations. Finally, we utilized the lung cancer data from the Finngen database R10 as our replication cohort. However, within the GWAS data for this lung cancer, there was a substantial disparity in case numbers versus control numbers across the four subtypes, leading to reduced statistical power for detecting associations between genes and phenotypes. This resulted in a relatively low replication rate between our discovery and replication phases. Thus, the causal relationships of the remaining genes with lung cancer require further scrutiny and investigation.

In conclusion, our MR analysis revealed a relationship between the HYKK (AGPHD1) gene and the risk of developing lung squamous cell carcinoma, consistent with prior randomized controlled trials and meta-analysis studies. This gene represents a promising therapeutic target for both prevention and treatment of lung squamous cell carcinoma, yet its functional mechanisms require further experimental investigation.

### Supplementary Information

Supplementary Material 1.

Supplementary Material 2.

## Data Availability

No datasets were generated or analysed during the current study.
